# Self-complementarity in adeno-associated virus enhances transduction and gene expression in mouse cochlear tissues

**DOI:** 10.1371/journal.pone.0242599

**Published:** 2020-11-23

**Authors:** Graham Casey, Charles Askew, Mark A. Brimble, R. Jude Samulski, Andrew M. Davidoff, Chengwen Li, Bradley J. Walters

**Affiliations:** 1 Program in Neuroscience, University of Mississippi Medical Center, Jackson, MS, United States of America; 2 Gene Therapy Center, University of North Carolina at Chapel Hill, Chapel Hill, NC, United States of America; 3 Department of Surgery, St. Jude Children’s Research Hospital, Memphis, TN, United States of America; 4 Department of Hematology, University College London Cancer Institute, London, United Kingdom; 5 Department of Pharmacology, University of North Carolina at Chapel Hill, Chapel Hill, NC, United States of America; 6 Department of Pediatrics, University of North Carolina at Chapel Hill, Chapel Hill, NC, United States of America; 7 Carolina Institute for Developmental Disabilities, University of North Carolina at Chapel Hill, Chapel Hill, NC, United States of America; 8 Department of Neurobiology and Anatomical Sciences, University of Mississippi Medical Center, Jackson, MS, United States of America; 9 Department of Otolaryngology-Head and Neck Surgery, University of Mississippi Medical Center, Jackson, MS, United States of America; Fudan University, CHINA

## Abstract

Sensorineural hearing loss is one of the most common disabilities worldwide. Such prevalence necessitates effective tools for studying the molecular workings of cochlear cells. One prominent and effective vector for expressing genes of interest in research models is adeno-associated virus (AAV). However, AAV efficacy in transducing cochlear cells can vary for a number of reasons including serotype, species, and methodology, and oftentimes requires high multiplicity of infection which can damage the sensory cells. Reports in other systems suggest multiple approaches can be used to enhance AAV transduction including self-complementary vector design and pharmacological inhibition of degradation. Here we produced AAV to drive green fluorescent protein (GFP) expression in explanted neonatal mouse cochleae. Treatment with eeyarestatin I, tyrphostin 23, or lipofectamine 2000 did not result in increased transduction, however, self-complementary vector design resulted in significantly more GFP positive cells when compared to single-stranded controls. Similarly, self-complementary AAV2 vectors demonstrated enhanced transduction efficiency compared to single stranded AAV2 when injected via the posterior semicircular canal, *in vivo*. Self-complementary vectors for AAV1, 8, and 9 serotypes also demonstrated robust GFP transduction in cochlear cells *in vivo*, though these were not directly compared to single stranded vectors. These findings suggest that second-strand synthesis may be a rate limiting step in AAV transduction of cochlear tissues and that self-complementary AAV can be used to effectively target large numbers of cochlear cells *in vitro* and *in vivo*.

## Introduction

Hearing is one of our primary senses and plays a key role in several vital functions including communication, learning and memory, and situational awareness. This highly evolved system is capable of distinguishing, amplifying, and transducing minute pressure waves into neurochemical signals that are ultimately perceived as sound. The speed, resolution, and sensitivity of the peripheral auditory system all reflect its exquisite functional capabilities and the evolutionary refinement that has led to its complex architecture. Despite the clear importance of this sensory system, there is still much to learn about its development, function, and response to environmental stressors and / or genetic mutations. As a collective result of these latter two etiologies, hearing loss is one of the most common long-term disabilities, affecting hundreds of millions of people worldwide, and costing billions of dollars each year to the U.S. healthcare system alone [1–3]. The most common type of hearing loss is sensorineural hearing loss (SNHL), which occurs as a result of either damage to the organ of Corti (OC) or the nerves that innervate it. While the majority of SNHL cases are believed to be caused by environmental insults such as noise and ototoxic drugs, a number of congenital causes for hearing loss have also been identified, including several that cause progressive SNHL which may be prevented or mitigated using viral gene therapy [4]. For these reasons it is important that a detailed understanding of genes as they relate to cellular functions of the inner ear be elucidated. One of the more popular vector-based tools for the modification of gene expression is the use of recombinant adeno-associated virus (AAV) [5]. AAV vectors have not only become a widely adopted and powerful research tool, but they also offer great promise for gene therapies to treat SNHL and other hearing and balance disorders [6, 7]. Unfortunately, AAV mediated gene transduction is not uniformly successful in the inner ear and a large degree of variability exists across published reports. Differences across AAV serotypes, across species or different mouse strains, across various ages or routes of administration, and simply from lab to lab are readily apparent [8–13]. Due to this fact, AAV mediated gene transduction may not always target an intended cell type with the desired efficiency, and often results in the use of high viral doses, which can be cytotoxic [5, 14–17]. It is therefore important to investigate means for improving AAV transduction efficiency in the sensory tissues of the inner ear [9, 18].

As AAV transduction is a multi-step process, transduction efficiency can be limited by several factors. First, AAV must bind to and be internalized by target cells typically using extracellular glycans or protein attachment factors [19, 20]. These include heparan sulfate proteoglycan, sialic acid, or integrins. Next, AAV must be trafficked through a productive infectious pathway to the nucleus, which for AAV serotypes 1, 2, 3, 5, 6, 7, 8, and 9 were recently identified to rely on the interaction with a trans-golgi associated transmembrane protein termed AAVR (KIAA0319L) [21]. During trafficking, AAV must avoid protease-mediated degradation and other unproductive pathways. Nuclear entry is critically dependent on externalization of the VP1/VP2 unique regions of the capsid proteins that contain a Phospholipase A domain and modular nuclear localization signals [22]. Finally, the capsid must be uncoated to release the single-stranded vector DNA and transduced cells must synthesize a second, complementary strand to the vector genome in order for the vector transgene to be transcribed [23]. Alternatively, multiple copies of positive and negative strand vector genomes can self-anneal, though this can be highly rate limiting as well [24]. In several different cell culture models, using cells not derived from the inner ear, a number of approaches have been shown to increase the efficiency of AAV transduction and transgene expression by targeting each of these potential bottlenecks [25, 26]. To the authors’ knowledge, these approaches have not been attempted in the inner ear and could therefore be used to optimize AAV transduction in the mouse cochlea, one of the primary models for the study of inner ear development and pathology. Accordingly, the first strategy attempted here was to increase AAV uptake into the cells. It has recently been reported that increased endocytosis of AAV particles that were purified in exosomes dramatically increased AAV transduction in inner ear tissues [27, 28]. Similarly, reports from other cell and tissue types suggest that packaging AAV in liposomes can enhance AAV transduction, presumably through a similar mechanism [29–31]. This can be accomplished through the use of cationic lipid complexes such as Lipofectamine reagents, which are widely documented as a tool for the transfection of macromolecules into eukaryotic cells, including cells in the mouse inner ear [32]. A second strategy for enhancing AAV transduction efficiency is to prevent degradation of viral particles through pharmacological inhibition of endoplasmic reticulum (ER) associated protease activity [26]. Use of the drug eyearestatin I (EERI), and other approaches to inhibit ER mediated degradation have been shown to significantly increase AAV transduction in several different cell types [26, 28]. Finally, transduction can be increased by promoting or bypassing second-strand synthesis which can be accomplished pharmacologically or via self-complementary vector design. By generating AAV that contains self-complementary DNA sequence, second-strand synthesis can be bypassed, and transduction efficiency significantly increased [33]. Pharmacologically, compounds that target the FK506 binding protein (FKBP52) and its interaction with heat shock protein 90 (HSP90) can alter the kinetics of second-strand synthesis. One such compound, the tyrosine kinase inhibitor tyrphostin 23 (Tyr23) has been demonstrated to augment AAV mediated gene transduction by inhibiting the phosphorylation of FKBP52, thus stabilizing its interaction with HSP90 and enhancing second-strand synthesis [25, 34]. Here, we tested the transduction efficiency of two different AAV serotypes (AAV1 and AAV2) in explanted cochleae from neonatal mice and attempted to enhance transduction and GFP reporter gene expression through the use of either (1) Lipofectamine 2000, (2) EERI, (3) Tyr23, or (4) self-complementary AAV vectors. Surprisingly, despite the demonstrated efficacy of all of these approaches in other cell types, only the use of self-complementary AAV demonstrated a significant increase in viral transduction and GFP expression. Subsequent to this finding, we injected neonatal mice with self-complementary and single stranded AAV2 vectors via the posterior semicircular canal, and observed similar increases in transduction and GFP expression *in vivo*. Additionally, we validated self-complementary AAV vectors for serotypes AAV1, AAV8, and AAV9, all of which demonstrated robust transduction and GFP expression in the organ of Corti, *in vivo*.

## Materials and methods

### Production, purification and quantification of recombinant adeno-associated virus vectors

Vectors for *in vitro* use were generated using either an AAV1 rep-cap plasmid (LT-RC02, a gift from Dr. Richard Mulligan, Harvard Medical School) or an AAV2 rep-cap plasmid (LT_AAVhelp2_2, made by John Gray at St. Jude Children’s Research Hospital) The same pHelper plasmid was used for both strains (Part No. 340202, VPK-400, Cell Biolabs) and either a single stranded transgene plasmid (pAAV-400 from Cell Biolabs) or a self-complementary transgene plasmid (pAVscCMV_GFP, made by John Gray at St. Jude Children’s Research Hospital, Addgene 32396) were both constructed to have a CMV promoter driving GFP expression. Using polyethylenimine (Polyplus), plasmids were transfected into 293T cells (ATCC, CRL-3216) that were cultured in Dulbecco’s modified Eagle medium (DMEM, Lonza) supplemented with 10% fetal bovine serum (FBS, HyClone) and 2 mM L-Glutamine (Corning). Post-transfection, cells were incubated at 37 ˚C and 10% CO_2_. Media was changed after 18 hours to serum-free DMEM + 2mM L-Glutamine, and the cells were incubated until 72 hours post-transfection. 293T cells were then scraped from plates, spun down in 50ml Falcon tubes at 3000 x g for 10 minutes and pellets were resuspended in 5ml PBS. Crude cell lysates were freeze-thawed at -80˚C five times and spun at 3000g for 10 minutes. Cell lysate supernatant was transferred to a 50ml conical tube and diluted with DMEM to a volume of 37.5 mL. 12.5ml 40% polyethylene glycol (PEG) was added to the solution and incubated at 4°C for 2 hours. AAV were pelleted by centrifugation at 3000 x g for 30 min. PEG-precipitated pellets were resuspended in 10mM Tris 10mM Bis Tris Propane (pH9, Millipore-Sigma) and then treated with benzonase (Millipore-Sigma) for 1hr at 37˚ C and transferred to a 1.5g/mL:1.3g/mL Cesium Chloride step gradient in 32 mL Thickwall ultracentifuge tubes (Beckmann Coulter) and spun at 24,600rpm in an ultracentrifuge at RT for 20 hrs (SW32Ti rotor). Full particles were extracted and dialysed in 10KDa cassettes (Thermo Fisher) in 1X PBS for 1 hour 3 times. Dialysed virus was concentrated in 100KDa centrifugal filter tubes to a volume of 0.25ml (Amicon Ultra-15, Millipore) in a benchtop centrifuge at 2,000 x g. Virus was resuspended in PBS to a total volume of 0.5ml and stored in aliquots at -80˚C. Purified AAV was titered by qPCR using serially diluted virus in ddH20 + 0.01% Pluronic F-68 (Gibco) and quantitated against linearised serially diluted plasmid standard. Forward CMV primer: ATATGCCAAGTACGCCCCCTATTGAC and Reverse CMV primer: ACTGCCAAGTAGGAAAGTCCCATAAGGTC, (analysis run on Applied Biosystems 7500).

AAV2/2 (AAV2) vectors for *in vivo* injections were produced by the Gene Transfer Vector Core, Grousebeck Gene Therapy Center at Massachusetts Eye and Ear Infirmary (https://www.vdb-lab.org/vector-core/). These vectors were designed to have the CMV promoter driving the expression of enhanced GFP (EGFP) and included a Woodchuck Hepatitis Virus Posttranscriptional Regulatory Element (WPRE) preceding the bovine growth hormone (BGH) polyA termination sequence. After purification, vectors were stored frozen in 1x PBS including 35mM NaCl and 0.001% PF68 until use.

AAV1, AAV8, and AAV9 vectors for *in vivo* injection were produced by the University of North Carolina Vector Core generated by the standard helper virus-free triple transfection method as previously described [35] using a pXX6-80 adenoviral helper plasmid, the self-complementary pTRs-CBh-eGFP-BGHpA transgene plasmid [36], along with RepCap pXR plasmids for serotypes 1, 2, 8, and 9 [37]. All vectors were packaged using the self-complementary ITR sequences from AAV serotype 2 [38]. Crude lysate containing AAV vectors was purified by ultracentrifugation using an iodixanol step gradient followed by anion exchange chromatography. Purified vector was dialyzed into PBS containing 5% D-sorbitol. Vector titers ranged from 2×10^12^ to 4×10^12^ gc/mL as measured by quantitative PCR using ITR2 recognizing primer sets (Forward primer: AACATGCTACGCAGAGAGGGAGTGG and Reverse Primer: CATGAGACAAGGAACCCCTAGTGATGGAG). Virus aliquots were stored at −80°C and thawed just prior to use.

### Animals and housing

For *in vitro* experiments, male and female mice of a mixed background strain (129S6-C57Bl/6) were housed as breeding pairs under a 12:12 light/dark cycle (6 am lights on and 6 pm lights off), in a temperature and humidity-controlled environment with free access to water and food (LabDiet Mouse Diet 5015). All procedures followed NIH guidelines for the care and use of laboratory animals and were approved by the Institutional Animal Care and Use Committee (IACUC) at the University of Mississippi Medical Center (protocol #1485). For *in vivo* experiments, both male and female C57BL6/J mice were used. All animal work was done according to protocols approved by the Institutional Animal Care and Use Committee at the University of North Carolina (protocols #15–086; #15–295).

### Organ of corti explant culture

Mice to be used for cochlear explants were collected between postnatal day 0 (P0) and P1. They were rapidly decapitated and their organs of Corti (OC) were microdissected in ice cold 10% Hank’s Basic Salt solution (HBSS, Gibco) with .05% HEPES (Hyclone) in ultrapure water (Invitrogen) set to a pH of 7.2. Culture plates (50 mm glass bottom dishes, MatTek) were warmed in the incubator at 37° C for 1 hour with a 100 μl mixture of Matrigel (Corning) and DMEM (Gibco) at a ratio of 6:100 by volume coating the center of the dish. After dissection, the tissue was transferred via micro dissecting curette into the Matrigel/DMEM mixture. Excess matrigel/DMEM mixture was then removed without exposing the tissue to the air, and tissue was left to adhere to the dish for 10 minutes before culture media was added. Culture media was DMEM supplemented with 4% FBS (Hyclone), 1% N2 supplements (R&D Systems), 1 nM apocynin (Selleckchem) and 10 mg/ml ampicillin sulfate (Fisher). Cultures were placed in a 37°C incubator (5% CO2) for 24 hours before receiving EERI, Tyrphostin 23, or fresh media.

### Treatment of AAV1 explant cultures

After 24 hour incubation, explants were treated with Eeyarestatin I (EERI; 10 μM Calbiochem cat. #324521), Tyrphostin 23 (T23; 100 μM Alfa Aesar cat. #J60308), or fresh media for 4 hours. Subsequently, the explant cultures were exposed to AAV1 which was diluted into the culture media at a final concentration of 1 x 10^11^ gc/mL. For the liposomal packaging group, Lipofectamine 2000 reagent (Invitrogen) in Opti-MEM media (Gibco) at a volume ratio of 2:25 was added to AAV-containing Opti-MEM media and incubated for 5 min at room temperature. Control AAV was mixed into Opti-MEM media without Lipofectamine 2000. These solutions were then mixed into the DMEM media as described above so that the final AAV concentration was 1 x 10^11^ gc/mL. Culture media was removed from the explanted tissues and replaced with AAV containing media. Explants were then incubated in AAV containing media for 48 hours at 37°C and then fixed in 4% paraformaldehyde for 3 hours at room temperature. Control treatment for AAV1 consisted of a single stranded CMV-GFP (ss-CMV-GFP), and ss-CMV-GFP AAV was also used in the EERI, Tyrphostin 23, and Lipofectamine 2000 groups. An additional experimental group AAV1 was incubated with self-complementary CMV-GFP AAV with no additional treatments. At least 4 cochlear explants were tested in each condition.

### Treatment of AAV2 explant cultures

Immediately after dissection explants were treated with Eeyarestatin I (EERI; 10 μM Calbiochem cat. #324521), Tyrphostin 23 (T23; 100 μM Alfa Aesar cat. #J60308), or fresh media for 4 hours. Subsequently, the explant cultures were exposed to AAV2 which were diluted into the culture media at a final concentration of 1 x 10^11^ gc/mL. For the liposomal packaging group, Lipofectamine 2000 reagent (Invitrogen) in Opti-MEM media (Gibco) at a volume ratio of 2:25 was added to AAV-containing Opti-MEM media and incubated for 5 min at room temperature. Control AAV was mixed into Opti-MEM media without Lipofectamine 2000. These solutions were then mixed into the DMEM media as described above so that the final AAV concentration was 1 x 10^11^ gc/mL. Culture media was removed from the explanted tissues and replaced with AAV containing media. Explants were then incubated in AAV containing media for 5 days at 37°C and then fixed in 4% paraformaldehyde for 1 hour at room temperature. Media changes were performed every other day. Control treatment for each AAV2 consisted of a single stranded CMV-GFP (ss-CMV-GFP), and ss-CMV-GFP AAV was also used in the EERI, Tyrphostin 23, and Lipofectamine 2000 groups. An additional experimental group was incubated with self-complementary CMV-GFP AAV2 with no additional treatments. At least 6 cochlear explants were tested in each condition.

### Immunostaining and imaging

To quantify GFP+ cells in the organ of Corti, hair cells were visualized using mouse anti-parvalbumin (1:1,000 overnight at 4° C, Sigma cat. #P3088) and goat anti-mouse conjugated to Alexa 568 fluorophore (1:1,000 for 1.5 hours at room temperature, ThermoFisher). Imaging was performed on a Nikon C2 confocal microscope under a 20X objective. NIS Elements Acquisition image acquisition software was used to capture z stack images of samples. Camera and laser settings were uniform for control groups, and experimental groups were imaged at the same or lower laser power and gain than control groups. All images were analyzed using ImageJ software. Images were coded and a blinded investigator counted GFP positive cells. A grid was overlaid on each image, with each square in the grid pattern demarcating 1,000 μm^2^. Each region counted was at least 10,000 μm^2^ in area and was composed of at least 10 contiguous 1,000 μm^2^ squares that bounded at least one parvalbumin positive hair cell. Counts were then averaged to create the total average count for the sample. Characteristics quantified included total number of GFP positive cells per 10,000 μm^2^ and total number of GFP positive HCs per 10,000 μm^2^.

### In vivo AAV injections

For the direct comparison of AAV2 vectors, neonatal mice (P1-P2) were injected via the posterior semicircular canal as has been previously described [14]. Briefly, mouse pups were anesthetized on ice, then the post-auricular area was cleaned with iodine and a small incision made using a sterile scalpel. Glass pipets were pulled using a PUL-1000 (World Precision Instruments) to create a ~20 μm tip diameter and broken with forceps to create a sharp point. The glass needles were loaded with either single stranded or self-complementary AAV2/2.CMV.EGFP.WPRE.bGH vectors at a concentration of 9.5 x 10^11^ gc/mL. Using the Nanoliter 2010 microinjector (World Precision Instruments), 690 nL of vector were injected into the posterior semicircular canal at a rate of 69 nL every 10–15 seconds. After each injection, totaling 6.56 x 10^8^ viral genomes (vg), the needle was removed, the incision site closed using Vetbond (3M), and animals were placed in a heated cage to recover before being returned to their home cage. 14 days after injection, mice were euthanized and temporal bones were collected and fixed in 4% paraformaldehyde for 3–4 hours at room temp, then stored in PBS at 4°C. Cochlear tissues were dissected out from the temporal bones in TBS, then immunostained using a rabbit anti-myosinVIIA antibody (Proteus Biosciences) at 1:300 dilution to label hair cells. After secondary antibody labeling (Goat anti-rabbit, Alexa568 conjugated, ThermoFisher), confocal z-stack images of samples were then acquired on a Zeiss LSM880 at 20X. GFP positive cells were quantified manually using ImageJ software.

For injection of AAV1, AAV8, and AAV9 vectors, neonatal mice, age P0-P3, were injected unilaterally via the round window membrane as previously described [13]. Briefly, vector was introduced into the inner ear using beveled glass microinjection pipettes. Pipettes were pulled from capillary glass on a P-2000 pipette puller (Sutter Instruments) and beveled (~20 μm tip diameter at a 28° angle) using a micropipette beveler (BV10, Sutter Instruments). Mice were transiently anesthetized by induction of hypothermia. The surgical site was disinfected by scrubbing with Betadine and wiping with 70% Ethanol in repetition three times. A post-auricular incision was made behind the pinna to expose the muscles and fascia of the neck. The facial nerve was followed to where it enters the skull and the cartilagenous tympanic bulla was identified. Trans-illumination was used to identify the round window through the bulla, and the glass pipette was driven through the bulla into the round window membrane for vector delivery. Approximately 1μl of virus was injected unilaterally at a rate of 0.5μl/min using a pneumatic microinjector (Pico-injector PLI-100A, Warner Instruments), and the skin incision was closed using a 6–0 monofilament suture (Ethicon). Mice were then returned to the warming pad for recovery. For each serotype, 3 to 4 animals were injected and their cochleae were harvested for histology 1–2 weeks post injection. Confocal images were collected of the entire cochlea under a 5X objective, while representative sample images from apical, middle, and basal turns were collected under a 20x objective and used to quantify the number of eGFP expressing inner and outer hair cells.

For imaging of *in vivo* AAV-GFP injected cochleae, the temporal bones were harvested and immediately perfused with 4% paraformaldehyde and fixed for 1hr on ice. The cochleae were then decalcified by submersion in 120mM EDTA for 24hrs to allow microdissection of the bone and isolation of the organ of Corti and membranous labyrinth. Whole mounted cochlear preparations were stained with Alexa-fluor 546 Phalloidin (Molecular Probes) at a 1:200 dilution to label filamentous actin of the reticular lamina and hair bundles. Organ of corti samples were mounted on glass slides and imaged using a Zeiss LSM-710 confocal microscope and images were captured at 10X-20X magnification. Native eGFP fluorescence from the AAV-GFP expression was imaged without immunohistochemical amplification, and hair cells were identified morphologically. Z-stack images were generated using Image-J (NIH).

### Statistics

For *in vitro* comparisons, and for each serotype (AAV1 or AAV2), GraphPad Prism software was used to conduct a one-way ANOVA with Dunnett’s correction for multiple comparisons. Results were considered significant when the adjusted p value was less than 0.05. For *in vivo* comparison, independent samples t-tests were conducted for each comparison for each cell type in each cochlear region and the Holm-Bonferroni method was used to adjust for multiple comparisons. Results were considered significant when the adjusted p value was less than 0.05.

## Results

### AAV2 transduction in cochlear explant cultures

**Fig 1:**
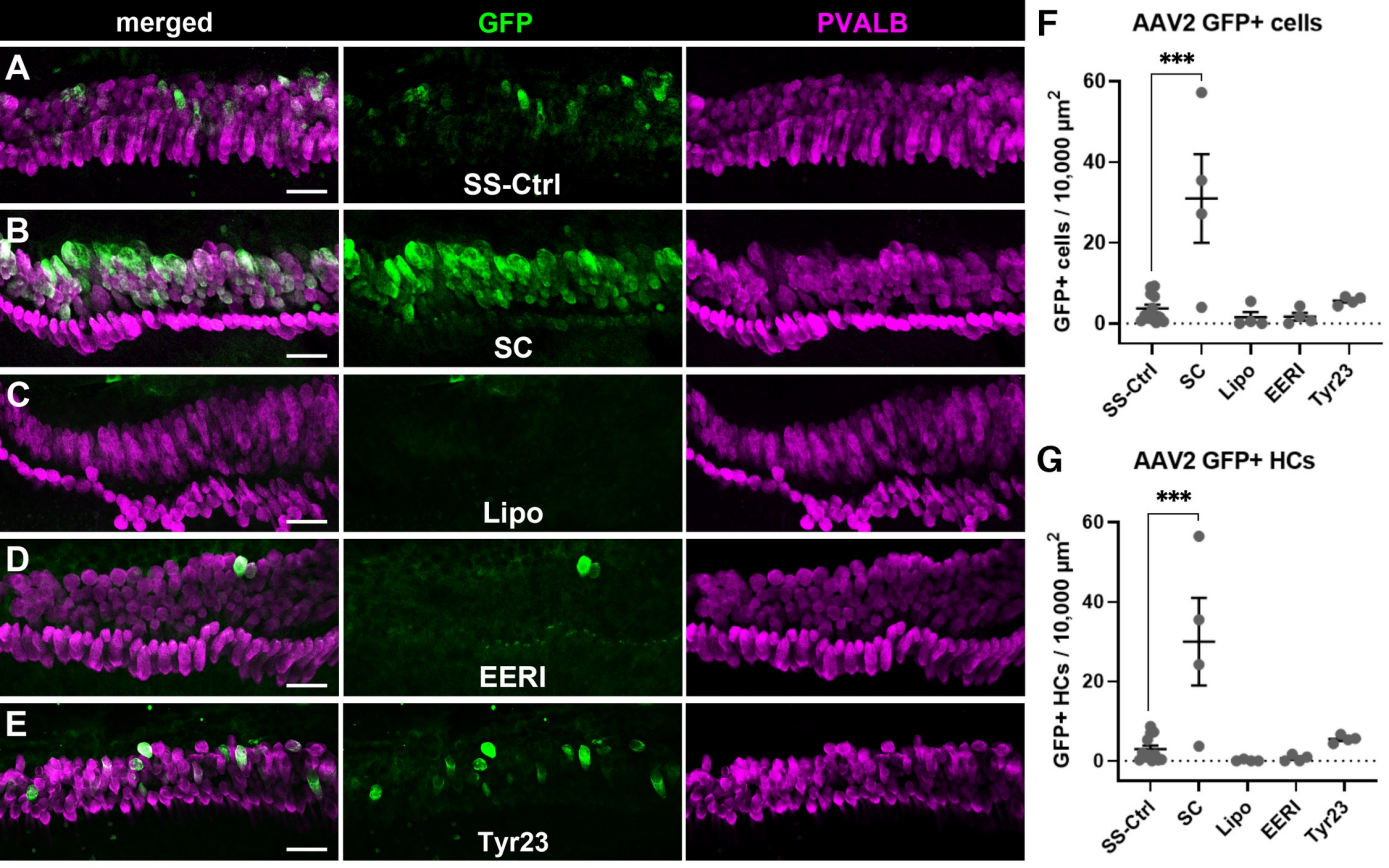
Self-complementarity increased transduction efficiency of AAV2-mediated GFP expression in the organ of Corti. (A) A representative image from a neonatal cochlear explant treated with ssAAV2-CMV-GFP (SS-Ctrl) with GFP shown in green and parvalbumin (PVALB) shown in magenta. (B) A representative image from a neonatal cochlear explant treated with scAAV2-CMV-GFP which shows a large number of cells in the organ of Corti as being GFP positive. (C–E) Show images from explants treated with ssAAV2-CMV-GFP virions that were either packaged with Lipofectamine 2000 reagent (C) or pretreated with EERI (D) or Tyr23 (E). Post-hoc comparisons of each treatment group to the control group showed a significant effect of self-complementarity (F) on total numbers of GFP positive cells in the organ of Corti, and similarly a significant effect of self-complementary AAV on the numbers of GFP positive hair cells specifically (G). Error bars represent ±1 SEM, *** denotes p < 0.001, and all scale bars = 20 μm.

Cochleae were explanted from P0-P1 mice and then treated with Lipofectamine 2000 (Lipo), EERI, Tyr23, or vehicle. Subsequent to liposome packaging or treatment, AAV2 particles containing CMV promoter-driven GFP that were either single-stranded (ssAAV2-CMV-GFP) or self-complementary (scAAV2-CMV-GFP) were applied to the tissue. After 2 days of AAV exposure, fresh media was applied and explants were maintained an additional 3 days in vitro (DIV). Total numbers of GFP-positive cells in the organ of Corti were quantified as well as GFP positive cells that co-expressed parvalbumin which is readily detectable in sensory hair cells but not supporting cells. ANOVAs for each of these measures for the AAV2 serotype revealed significant main effects of treatment for both the total numbers of GFP positive cells (F_(4, 23)_ = 9.511, p = 0.0001) and for the numbers of GFP positive hair cells (F_(4, 23)_ = 9.701, p < 0.0001). Post-hoc comparisons suggest that this significant main effect was driven by a significant increase in the numbers of cells transduced by the self-complementary virus (Fig 1). Explants that received scAAV2-CMV-GFP as compared to those that received ssAAV2-CMV-GFP had significantly more GFP positive cells in the sensory epithelium (31.0 compared to 3.69 GFP-positive cells per 10,000^2^ μm, p = .0001, Fig 1F) as well as significantly more GFP positive hair cells as defined by co-expression with parvalbumin (29.97 compared to 2.92 for control GFP-positive HCs per 10,000^2^ μm, p < 0.0001 Fig 1G). However, none of the other treatments resulted in GFP expression that differed significantly from the single stranded AAV2 controls. Neither lipofection nor treatment with EERI or Tyr23 showed any effect on total numbers of GFP positive cells (1.5, 1.67, and 5.67 GFP-positive cells per cells per 10,000^2^ μm, p = 0.9742, p = 0.9806, and p = 0.9820, respectively) nor did they reveal any effect on the numbers of GFP positive hair cells following AAV2 treatment (0.125, 0.667, and 5.50 GFP-positive HCs per cells per 10,000^2^ μm, p = 0.9377, p = 0.9700, and p = 0.9527, respectively).

### AAV1 transduction in cochlear explant cultures

**Fig 2:**
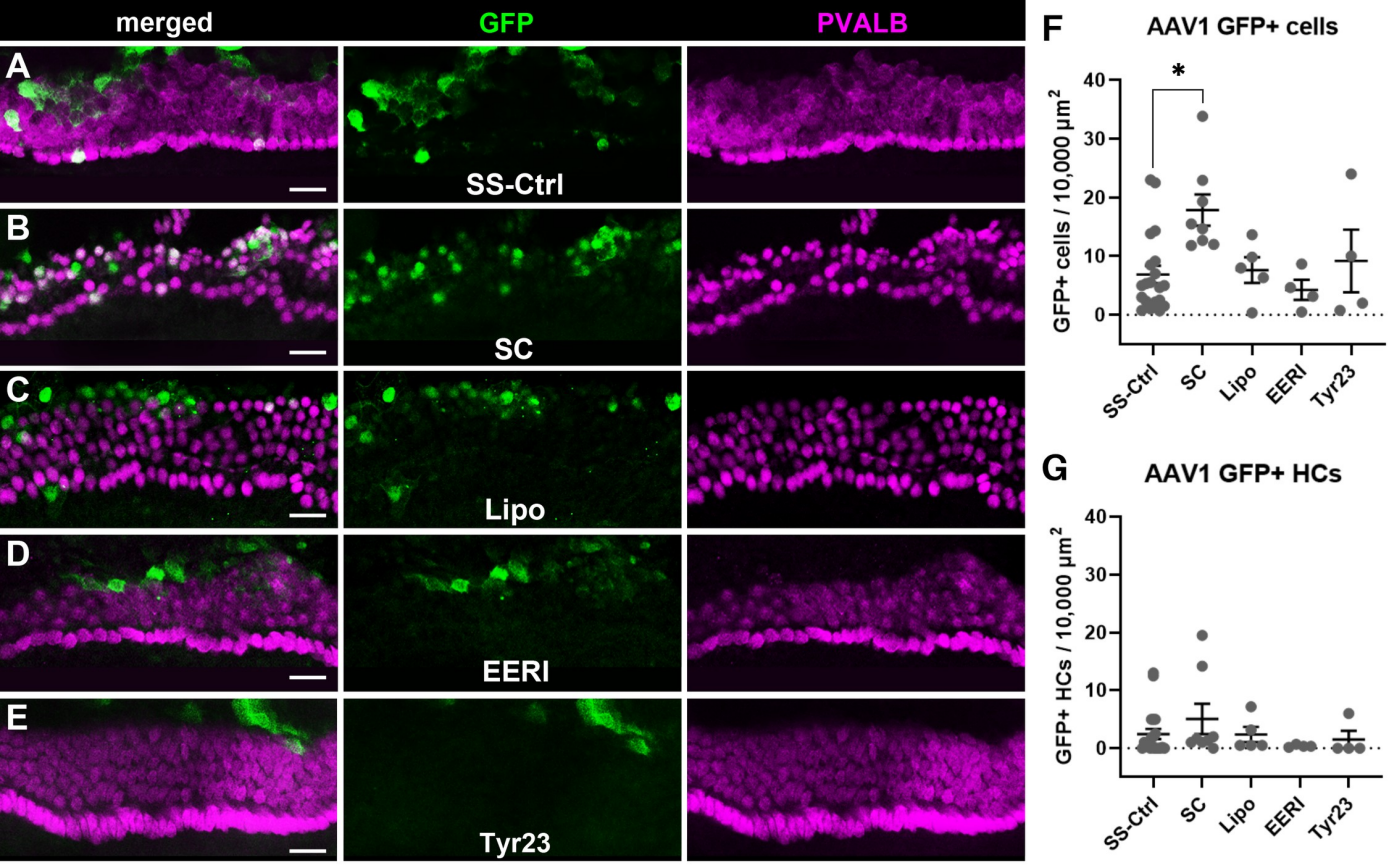
Self-complementarity increased transduction efficiency of AAV1-mediated GFP expression in the organ of Corti. (A) A representative image from a neonatal cochlear explant treated with ssAAV1_CMV_GFP (SS-Ctrl) with GFP shown in green and parvalbumin (PVALB) shown in magenta. (B) A representative image from a neonatal cochlear explant treated with scAAV1-CMV-GFP. (C–E) Show images from explants treated with ssAAV1-CMV-GFP virions that were either packaged with Lipofectamine 2000 reagent (C) or pretreated with EERI (D) or Tyr23 (E). Post-hoc comparisons of each treatment group to the control showed a significant effect of self-complementarity (F) on total numbers of GFP positive cells in the organ of Corti, but no effect of any treatment condition on the numbers of GFP positive hair cells (G). Error bars represent 1 SEM, * denotes p < 0.05, and all scale bars = 20 μm.

Cochleae were explanted from P0-P1 mice, grown overnight in culture, and then treated with Lipo, EERI, Tyr23, or vehicle. After liposome packaging or treatment, AAV1 particles containing CMV promoter-driven green fluorescent protein (GFP) that were either single-stranded (ssAAV1-CMV-GFP) or self-complementary (scAAV1-CMV-GFP) were applied to the tissue. Total numbers of GFP-positive cells in the organ of Corti were quantified as well as GFP positive hair cells that co-expressed parvalbumin. ANOVAs for each of these measures revealed a significant main effect of treatment (F_(4, 36)_ = 2.694, p = 0.0063) for total GFP+ cells, but no main effect of treatment for GFP positive hair cells (F_(4, 36)_ = 0.905, p = 0.4712). Similar to what was observed with AAV2, the main effect of treatment appears to be due solely to the enhanced transduction seen in the self-complementary group (Fig 2). Post-hoc comparisons revealed that explants that received scAAV1-CMV-GFP as compared to those that received ssAAV1-CMV-GFP had significantly more GFP positive cells in the sensory epithelium (17.8 compared to 6.89 GFP-positive cells per 10,000^2^ μm, p = 0.002, Fig 2F). However, self-complementary AAV1 differed slightly from self-complementary AAV2 in that it did not significantly increase the numbers of GFP and parvalbumin double positive hair cells specifically (5.08 compared to 2.45 GFP-positive HCs per 10,000^2^ μm, p = 0.512, Fig 2G). Again, neither lipofection nor treatment with EERI or Tyr23 showed any effect on total numbers of GFP positive cells (7.63, 4.25, and 9.19 GFP-positive cells per 10,000^2^ μm p = 0.9990. p = 0.9230, and p = 0.9515, respectively).

### AAV2 transduction in neonatal mouse cochleae in vivo

**Fig 3:**
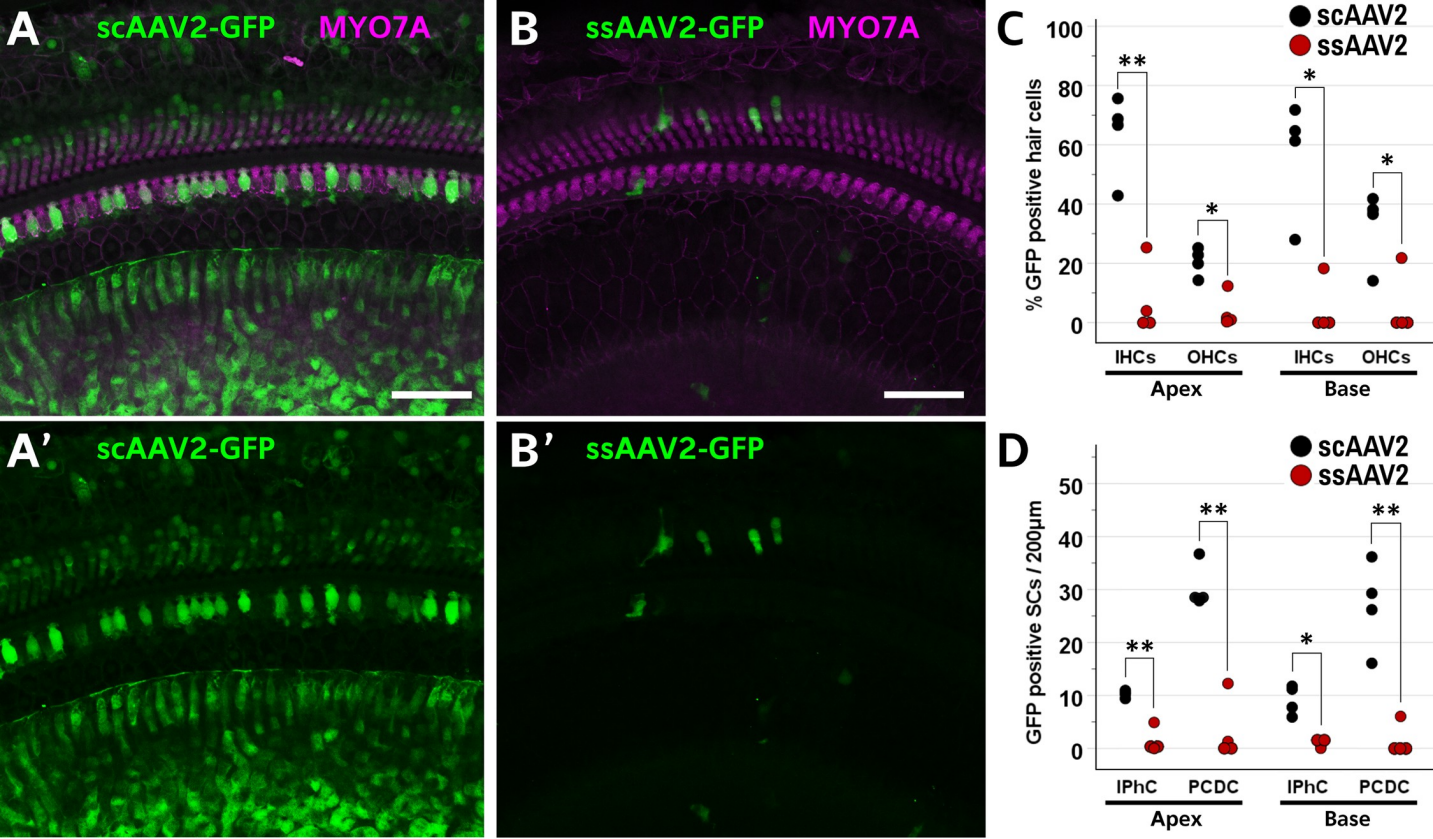
Self-complementary AAV2 transduces more cochlear cells than single stranded vectors following inner ear injections *in vivo*. (A, A’) Numerous cells in the cochlear duct, including supporting cells and MYO7A positive hair cells (magenta) express GFP (green) following semicircular canal injection of a self-complementary AAV2 vector (scAAV2-GFP). (B, B’) In contrast, injection of a single stranded AAV2 vector (ssAAV2-GFP) at the same dose (6.6 x 10^8^ vg) resulted in only a small number of cells expressing GFP. (C) Treatment with scAAV2-GFP (black dots) results in significantly more GFP positive inner hair cells and more GFP positive outer hair cells in both the apical and basal turns of the cochlea as compared to ears treated with ssAAV2-GFP (red dots). (D) Treatment with scAAV2-GFP (black dots) results in significantly more GFP positive inner phalangeal cells (IPhC) and significantly more GFP positive pillar and Deiters cells (PCDC) in both the apical and basal turns of the cochlea as compared to ears treated with ssAAV2-GFP (red dots). Scale bars represent 50 μm. Asterisks indicate significance of corrected p values: *p < 0.05, **p < 0.01.

A direct comparison of *in vivo* transduction of self-complementary versus single stranded AAV2-GFP vectors was undertaken via posterior semicircular canal injections in neonatal mice. Both the single-stranded and self-complementary AAV2 vectors were designed with the CMV promoter, EGFP coding sequence, and a WPRE element preceding the bGH polyA terminator sequence. Vectors were injected in the animals’ right ears and equal titers and equal volumes were used for each vector so that each animal received a total injected dose of 6.6 x 10^8^ vg regardless of whether they received the single stranded or self-comlementary vector. For this experiment, GFP positive hair cells were counted, as well as GFP positive supporting cells, and images were analyzed separately depending on whether they came from the apical or basal halves of the cochleae, representing the lower and higher frequency regions, respectively. Specifically, counts were made for inner hair cells (IHCs), outer hair cells (OHCs), inner phalangeal cells (IPhCs), and pillar and Deiters cells (PCDCs). In both the apical and basal turns, and for all of the cell types counted, the numbers of GFP positive cells were significantly higher in the cochleae from mice treated with the self-complementary AAV2 vector as compared to the single-stranded vector (Fig 3). Indeed, when collapsed across both the apical and basal turns, the average percentages of IHCs and OHCs that were GFP positive in the scAAV2 group were 59.9% and 26.5% compared to only 4.6% and 5.6% in the single stranded group. Similarly, there were more GFP positive IPhCs and PCDCs in the self-complementary AAV2 group (9.7 and 28.6 per 200 μm) than in the single stranded group (0.9 and 3.0).

### In vivo administration of self-complementary vectors for AAV serotypes 1, 8, and 9

**Fig 4:**
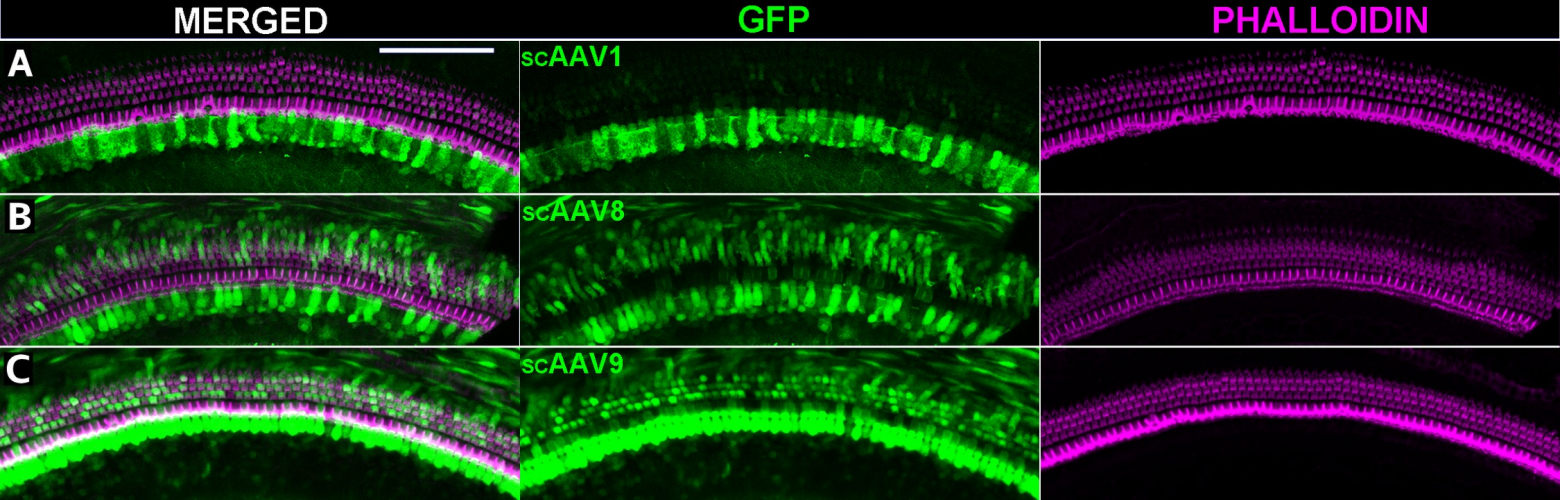
Self-complementary AAV vectors administered via round window membrane injection demonstrate robust GFP expression *in vivo*. (A-D) Representative confocal images of the organ of Corti 1–2 weeks after neonatal injection with self-complimentary vectors encoding eGFP driven by a CBh promoter packaged into AAV serotype capsids 1 (A), 8 (B), or 9 (C). Merged images are separated into their individual channels with AAV vector derived eGFP in the middle column (green) and Alexa-546 conjugated phalloidin in the right column (magenta). Mice were injected with 1μl of vector via the round window membrane at age P0-P3 with a total dose of 2–4 x 10^9^ viral genomes per ear. Scale bar = 100 μm.

To see if self-complementary AAV vectors of other serotypes can efficiently transduce cochlear cells *in vivo*, sc-AAV vectors for serotypes 1, 8, and 9 were injected into neonatal mouse inner ears via the round window. These AAV vectors contained the compact ubiquitous CBA hybrid promoter [39] (CBh) driving expression of enhanced green fluorescent protein (scAAV-CBh-eGFP). Total vector dose per ear was approximately 2 x 10^9^ vector genomes (vg) for AAV1, AAV2, and AAV8 while AAV9 was delivered at a dose of 4 x 10^9^ vg. Representative images reveal highly efficient transduction and expression of the eGFP for all three serotypes tested (Fig 4). Self-complementary vectors demonstrated robust *in vivo* transduction efficiency for serotypes 1, 8, and 9. Remarkably, scAAV9 transduced nearly all inner (93.8%) and outer (81.7%) hair cells along the cochlear duct, which has not previously been observed for ssAAV9. scAAV8 also transduced both inner (89.3%) and outer (49.8%) hair cells, but about half as many outer hair cells as scAAV9, though this may be due to the titer of scAAV8 being half of what was used for scAAV9 in these experiments. Conversely, scAAV1 transduced predominantly inner hair cells (78.1%) while sparing outer hair cells (9.2%). Our results support published *in vivo* reports that consistently demonstrate the cellular tropism of AAV1 as predominantly targeted toward inner hair cells [5, 8–10, 13, 28, 40].

## Discussion

Recently, several reports have highlighted the promise of AAV to be used as vectors for gene therapies that could potentially offset mutations that cause SNHL [7, 40–44]. In addition to the preservation or restoration of hearing in these animal models of congenital hearing loss, AAV vectors have proven invaluable in a number of studies examining the development and survival of cochlear cells, and their use has contributed significantly to our understanding of inner ear development and function [6, 7, 42, 45]. Furthermore, it is likely that AAV will continue to be adopted for many inner ear studies, including attempts to regenerate cochlear hair cells, a strategy that could potentially restore hearing in the majority of SNHL cases. Given this widespread adoption of AAV, the demonstrated importance to auditory research, and the potential importance to future clinical treatments to prevent or reverse SNHL, it is important to further our understanding of and approaches to the use of AAV in the inner ear.

However, AAV transduction efficiency in the inner ear can be variable. Much of this is due to AAV serotypes which have different binding and internalization efficiencies in different cell types [8, 9, 46]. Indeed, synthetic or reverse-evolved serotypes have recently been reported to dramatically increase transduction efficiency in the mouse inner ear, particularly *in vivo* [9, 14]. However, there are also multiple other factors that may affect AAV transduction efficiency in the inner ear. These range from the methods of preparation, purification, and storage of AAV, the volume or route of administration, the species or animal strain being used, and the age of the animal at the time of viral administration. Other factors that can influence AAV efficiency include choice of promoter, RNA stabilization elements such as the WPRE and polyA termination sequences utilized, as well as the method used to quantify viral particles and the vector genome titers at which the AAV is used. Many of these variables are determined by the nature of the research question being posed or the clinical outcome desired and therefore it becomes important to look for ways that might more generally improve AAV transduction efficiency so that investigators can choose the serotype, experimental model, preparation and purification methods, and genetic design that best suit their aims. Furthermore, the hair cells in the sensory organs of the inner ear are extremely sensitive and die in response to a number of different insults, included among them, high viral vector doses [14, 47, 48]. Thus, if methods for improving efficiency can be elucidated, they may ultimately permit the use of lower vector doses, which may decrease the ototoxic potential of certain AAV serotypes.

Here we tested a number of methods for the improvement of AAV transduction efficiency that have been previously established in cell culture systems, but have not been tested in the inner ear. These included lipofection, treatment with EERI or Tyr23, and the use of self-complementary rather than single-stranded vectors. Of these, only self-complementary vectors, led to significant increases in AAV transduction and GFP expression in our *in vitro* experiments. These findings, which apply across two different serotypes, suggest that self-complementary vector design may be a broadly applicable tool for increasing AAV transduction in cochlear tissue. While one potential drawback to the use of self-complementary AAV is that their maximum packaging capacity is limited (~2.4 kb), it is worthwhile to note that the coding sequence of many known deafness causing genes are small and wild type copies of these genes could still be packaged into self-complementary vectors for gene replacement (Table 1). Furthermore, while the generally accepted size limit for self-complementary AAVs is around 2.4 kb, some reports suggest that packaging of self-complementary genomes of 3kb or larger may be possible [49]. Also, novel AAV approaches like intein-mediated protein trans-splicing may allow for the use of scAAV for larger GOIs if increases in efficiency resulting from self-complementarity can allow for dose reductions greater in magnitude than the number of different AAV vectors that need to be combined [50].

**Table 1:** 

Locus	Size (CDS)	Gene/protein	Accession #
*USH1G*	1386 nt	*USH1G* / SANS	NM_173477.5
*USH3A*	738 nt	*CLRN1* / clarin-1	NM_001195794
*USH3B*	1530 nt	*HARS* / histidyl-tRNA synthetase	NM_002109.6
*PHYH*	1017 nt	*PHYH* / phytanoyl-CoA 2-hydroxylase	NM_006214
*TIMM8A*	294 nt	*TIMM8A* / translocase of inner mitochondrial membrane 8A	NM_004085
*CLDN9*	654 nt	*CLDN9* / Claudin-9	NM_020982.4
*GATA2*	1443 nt	*GATA2* / GATA binding protein 2	NM_001145661.2
*GATA3*	1335 nt	*GATA3* / GATA binding protein 3	NM_001002295.2
*ELMOD1*	1005 nt	*ELMOD1* / ELMO domain containing 1	NM_018712.4
*DFNA25*	1770 nt	*SLC17A8* / Vesticular glutamate transporter 3	NM_139319.3
*DFNB1*	681 nt	*GJB2* / gap junction protein B2 (connexin 26)	NM_004004.6
*DFNB6*	471 nt	*TMIE* / transmembrane inner ear expressed protein	NM_147196.2
*DFNB8*	1363 nt	*TMPRSS3* / transmembrane protease serine 3	NM_024022.3
*DFNB15*	939 nt	*GIPC3* / GIPC PDZ domain containing family member 3	NM_133261.3
*DFNB25*	873 nt	*GRXCR1* / Glutaredoxin Domain-containing Cycsteine-rich Protein 1	NM_001080476.2
*DFNB29*	720 nt	*CLDN14* / claudin 14	NM_144492.3
*DFNB35*	1527 nt	*ESRRB* / estrogen related receptor beta	NM_004452.3
*DFNB42*	1641 nt	*ILDR1* / immunoglobulin like domain containing receptor 1	NM_001199799.2
*DFNB48*	564 nt	*CIB2* / calcium integrin binding protein 2	NM_006383.4
*DFNB49*	1677 nt	*TRIC (MARVD2)* / tricellulin (marvel domain containing protein 2)	NM_001038603.3
*DFNB63*	876 nt	*LRTOMT* / leucine-rich transmembrane O-methyltransferase	NM_001145309.3
*DFNB59*	1059 nt	*PJVK* / pejvakin	NM_001042702.4
*DFNB66/67*	660 nt	*LHFPL5* / LHFP-like protein 5	NM_182548.4
*DFNB73*	963 nt	*BSND* / barttin	NM_057176.3
*DFNB74*	579 nt	*MSRB3* / methionine sulfoxide reductase B3	NM_198080.4
*DFNB76*	1215 nt	*SYNE4* / spectrin repeat-containing nuclear envelope	NM_001039876.3
*DFNB86*	1680 nt	*TBC1D24* / TBC1 domain family member 4	NM_001199107.2
*DFNB88*	1146 nt	*ELMOD3* / ELMO domain containing 3	NM_001135021.2
*DFNB91*	1131 nt	*SERPINB6* / serpin B6	NM_004568.5
*DFNB93*	681 nt	*CABP2* / calcium binding protein 2	NM_001318496.2
*DFNB94*	1434 nt	*NARS2* / asparaginyl-tRNA synthetase, mitochondrial	NM_024678.6
*DFNB101*	747 nt	*GRXCR2* / glutaredoxin domain-containing cysteine-rich protein 2	NM_001080516.1
*DFNB103*	1233 nt	*CLIC5* / chloride intracellular channel 5	NM_001114086.2
*DFNB107*	786 nt	*WBP2* / WW domain binding protein 2	NM_012478.4

The significant increases in GFP positive cells in response to scAAV1 and scAAV2 as compared to ssAAV1 and ssAAV2 suggest that inexpedient second-strand synthesis or self-annealing limit AAV mediated gene transduction in the sensory epithelium of the inner ear. While second-strand synthesis has been shown to be a limiting factor in several other cell types, there are some reported instances where self-complementarity did not enhance transduction, thus it was necessary to test this directly in cochlear tissue [33, 51, 52]. Indeed, all of the treatments tested herein have been shown to improve AAV transduction in other cell types, however, only self-complementary vector design had an effect in the cochlea. This is particularly interesting in the case of Tyr23 treatment, which should also have enhanced second-strand synthesis, but did not elicit any increases in GFP-positive cells in either AAV1 or AAV2 treated cultures. In some cell types, phosphorylation of tyrosine residues on FKBP52 has been shown to inhibit AAV second-strand synthesis in the host cells [25, 35]. Tyr23 is an inhibitor of protein tyrosine kinases and Tyr23 treatment has been shown to dephosphorylate FKBP52 leading to increased second strand synthesis [25]. Thus, the results presented here suggest that FKBP52 phosphorylation may not be the primary mechanism by which cochlear cells are preventing second-strand synthesis. However it is also possible that the Tyr 23 treatment did not adequately dephosphorylate FKBP52 in the cochlear cells. This could be due to the presence of tyrosine kinases that are not targeted by Tyr23 in neonatal mouse cochleae, or due to a lack of the appropriate phosphatases to dephosphorylate FKBP52 in the absence of its phosphorylating kinase. Indeed, Tyr23 is a fairly specific inhibitor of EGFR tyrosine kinase, and may not target casein kinase II which has also been shown to phosphorylate FKBP52 [35, 53, 54]. Additionally, neither lipofection of the AAV capsids nor pretreatment of the tissue with EERI seemed to increase transduction rates. Combined with the fact that self-complementary AAV showed robust GFP expression, the lack of effect for these other treatments suggests that vector entry and ER-associated protein degradation may not be primary factors limiting AAV efficiency in mouse cochlear explants. However, the current experiments cannot completely rule out that these factors can reduce transduction efficiencies in these cells. Indeed, the successes of exosome mediated AAV transduction and of synthetic AAV capsids (e.g. Anc80L65) suggest that cellular uptake is a limiting factor for AAV transduction in mouse cochleae [9, 28]. Thus it is possible that means other than those tested here are more efficacious in promoting AAV internalization, and it is also possible that the rate limiting step of second-strand synthesis coupled with the short time frame allowable by cochlear explant survival *in vitro* may have masked positive effects of Lipofectamine or EERI that might be observable over longer periods of time. Future experiments therefore will aim to test a combination of treatments to determine whether methods for targeting cellular uptake and/or evasion of ER-mediated degradation can further enhance the efficacy of self-complementary AAV.

Finally, buoyed by the *in vitro* results, and the potential for using self-complementary AAV to deliver therapeutically relevant GOIs, we tested self-complementary AAV *in vivo*, via posterior semicircular canal injections and by round window injections in neonatal mice. The results of these experiments confirmed greater transduction rates for self-complementary AAV2 vectors as compared to single stranded vectors, and demonstrate that self-complementary AAV of several different serotypes can induce robust GOI expression in the organ of Corti. The results here, summarized in Table 2 along with data from other publications using ss and sc AAV vectors in the inner ear, demonstrate that these self-complementary AAV vectors, when delivered at doses between 6 x 10^8^ and 6 x 10^9^ vg, are effective for transducing cochlear hair cells and in some cases surrounding supporting cells with very high efficiency [8, 9, 13, 28, 36, 40]. Indeed, our own previous experiments (same experimenter) [9, 13] suggest that a dose of ssAAV1 greater than 1 x 10^10^ vg per cochlea is required to achieve similar levels of GOI expression as scAAV1 at 6 x 10^9^ vg per cochlea. This notion that sc vectors can allow for reduced viral doses is supported by our direct comparison of ss and scAAV2 in vivo, where the low dose of 6.6 x 10^8^ vg for the sc vector was able to elicit fairly robust GFP expression in cochlear hair cells and supporting cells, while little to no GFP was observed with ssAAV2 at the same dose. Moreover, we report near complete transduction of all cochlear hair cells using scAAV9 at a dose of 4 x 10^9^ vg per neonatal cochlea, a phenomenon only observed with engineered capsids such as Anc80L65 or AAV2.7m8 [9, 14]. Additionally, although it is difficult to transduce outer hair cells with high efficiency using ssAAV, it appears here that scAAV vector genomes delivered by serotype 8, or 9 capsids are able to transduce a significant number of outer hair cells at a relatively low dose (2 x 10^9^ or 4 x 10^9^ vg). This could suggest that second-strand synthesis in outer hair cells is rate limiting, that vector entry into inner hair cells is much greater than for outer hair cells for most serotypes, or a combination of these two explanations. Finally, it is also important to note that critical aspects of tropism may be preserved or enhanced when using self-complementarity rather than capsid alteration as a strategy to increase AAV transduction. For example, while synthetic capsids such as Anc80L65 and AAV2.7m8 greatly enhance viral transduction in the cochlea, they increase transgene expression not only in inner and outer hair cells, but in a number of other cell types as well [9, 14]. Here, scAAV1-GFP vectors were shown to preserve their tropism for inner hair cells, particularly *in vivo*, suggesting they could potentially be more useful for ectopic expression of genes related to inner hair cell function specifically (e.g. *SLC17A8* a.k.a. *VGLUT3*). Thus certain self-complementary AAV vectors may possess an advantage over those with synthetic capsids when cell specificity is critical. Further study will help to determine the extent to which self-complementary AAV may enhance transduction efficiencies in the inner ear, the extent to which serotypical tropism may be preserved, or if self-complementarity may allow for dose reductions of recombinant AAV vectors across natural and synthetic serotypes without sacrificing therapeutic effect. In conclusion, as has been demonstrated in many other cell types and systems, the experiments here demonstrate that self-complementary AAV enhances transduction of GOIs in mouse cochlear cells and may therefore prove useful for genetic experiments and therapeutic approaches in the inner ear.

**Table 2:** 

Study	Vector format	AAV1		AAV2		AAV8		AAV9	
		IHC%	OHC%	IHC%	OHC%	IHC%	OHC%	IHC%	OHC%
This study	scAAV (6E8 – 4E9 vg)	78.1	9.2	59.9	26.5	89.3	49.8	93.8	81.7
György et al. [41]	scAAV (5E9 vg)	~88	~25						
Landegger, Pan, Askew et al. [9]	ssAAV (6E9–1.3E10 vg)	<70	<5	<5	<10	<20	<5	--	--
Shu et al. [8]	ssAAV (2E8–1.4E9 vg)	13.6	13.4	11.4	33.3	18.7	14.2	16.2	0
